# Pressure ulcer prevention practices and associated factors among nurses in public hospitals of Harari regional state and Dire Dawa city administration, Eastern Ethiopia

**DOI:** 10.1371/journal.pone.0243875

**Published:** 2020-12-15

**Authors:** Asmare Getie, Amsalu Baylie, Agegnehu Bante, Biftu Geda, Firehiwot Mesfin

**Affiliations:** 1 School of Nursing, College of Medicine and Health Sciences, Arba Minch University, Arba Minch, Ethiopia; 2 School of Nursing, College of Medicine and Health Sciences, Bahir Dar University, Bahir Dar, Ethiopia; 3 School of Public Health, College of Health and Medical Sciences, Haramaya University, Harar, Ethiopia; University Magna Graecia of Catanzaro, ITALY

## Abstract

**Introduction:**

Pressure ulcer is one of the major challenges in hospitals; which endanger patient safety, prolonging hospital stay and contributed to disability and death. Data regarding to pressure ulcer prevention practice are very important to take action. However in Ethiopia, there are limited researches that have been conducted and there is clearly paucity of information on this regard. Hence, this study aimed to assess pressure ulcer prevention practice and associated factors among nurses in public hospitals of Eastern Ethiopia.

**Methods:**

A cross-sectional study was conducted among randomly selected 422 nurses who were working in the public hospitals of Eastern Ethiopia. Data were collected from the 1^st^ February to the 1^st^ March 2018 using pretested structured self-administered questionnaire and observational checklist. The collected data were entered into EpiData version 3.1 and exported to SPSS version 22.0 for analysis. Bivariable and multivariable logistic regression with crude and adjusted odds ratios along with the 95% confidence interval was computed and interpreted accordingly. Pressure ulcer prevention was determined based on mean calculation; a result above the mean value was categorized as good pressure ulcer prevention practice, and a P-value of <0.05 was considered to declare a result as statistically significant.

**Results:**

In this study 51.9% (95% CI: 47.1%, 56.4%) of nurses were reported that they have good pressure ulcer prevention practice. On observation 45.2% of nurses were practicing proper pressure ulcer prevention activities. Pressure ulcer prevention practice were statistically associated with nurses with bachelor degree and above qualification level (AOR = 1.7, 95% CI: 1.02, 2.83), availability of pressure-relieving devices (AOR = 2.2, 95% CI: 1.34, 3.63), being satisfied with their job (AOR = 1.65, 95% CI: 1.09, 2.52) and good knowledge (AOR = 2.3, 95% CI: 1.48, 3.55).

**Conclusions:**

In this study the self-reported practice and results from observation was substantially low. Continuing education and training should be considered for nurses to enhance their practice regarding pressure ulcer prevention practice.

## Introduction

Pressure ulcers (PU) are lesion or injury to the skin or underlying tissues resulting from unrelieved pressure, shear, friction, or a combination of all these, usually over a bony prominence that may result in tissue death. It resulted from tissue compression between a bony prominence and an external surface for a prolonged period of time [1]. The consequences of pressure-induced skin injury range from non blanchable erythema of intact skin to deep bone [2]. The ulcer imposes a significant burden not only on the patient, but the entire health care system [3]. Pressure ulcers occur across all health care settings, with the highest incidence in the hospital. More recent data, however, recognized that the incidence of PU differs by care area, with patients in intensive care units, medical and surgical wards are at high risk of development of pressure ulcer [4, 5].

People with medical conditions that limit their ability to change positions or those who spend most of their time in bed or chair are mostly at risk of pressure ulcers. The elderly, patients with spinal cord injury and persons who are sedated from trauma or surgery are mainly at risk of developing pressure ulcer [6]. But, any person at any age could potentially develop a pressure ulcer if they were exposed to sustained unrelieved pressure, friction and shear forces for prolonged period of time [6, 7]. Peripheral vascular diseases, diabetic mellitus, smoking, prolonged immobility, poor nutritional status, incontinency, impaired sensation, use of steroids and aging, pressure, shear, friction, and moisture are considered as the factors which contributed to the development of pressure ulcers. Nurses’ knowledge and practice are also recognized as extrinsic factors for pressure ulcer formation [8–10].

Pressure ulcers are a largely preventable patient safety problem if appropriate interventions are implemented early and they are considered as an indicators to measure quality of nursing care and patient safety in the health care setting [11]. Pressure ulcers remain a severe and potentially life-threatening problem across all health care settings around the world. According to NPUAP report in 2017, showed that nearly 2.5 million patients develop PU every year and 60,000 patients died due to complication related to PU each year when it is not properly managed. In African, PUs is a common devastating complication among hospitalized patients and it affects 13. 84% patients in Nigeria and 16.8% in Ethiopia [12, 13].

Different studies were conducted in different part of the world to assess pressure ulcer prevention practice among nurses and the result revealed that pressure ulcer prevention practice was not adequate [14, 15]. Similarly in Ethiopia the proportion of nurses who had good practice towards pressure ulcer ranged from 48.4% to 67.3% [6, 16]. Pressure ulcers have been identified as a common and worldwide health problem that continues to cause of much discomfort and pain for patients and also lead to decreased quality of life, delayed healing, increase patient’s hospital stay, reduced performance, contributed to disability and death [16]. The financial expenditure for health care for patients who have developed a PU ranges from $750 million to greater than $1 billion [17].

Poor pressure ulcer prevention practice increases the incidence and prevalence of complications associated with PU in most healthcare settings. So, preventing pressure ulcers has become a key focus of many healthcare facilities in the world and it is a vital part of nursing care. Even though nurses make prevention as part of their routine care, several studies revealed that shortage of supplies for pressure ulcer prevention, heavy work load/ lack of staff, patient’s condition, lack of pressure ulcer related knowledge and job satisfaction were the identified barriers that hinder to carrying out appropriate pressure ulcer prevention practice [18]. Even though different researches were done on nurses’ practice towards pressure ulcer prevention globally, most of the researchers depended on self-administered response which could have limitation due to bias. Therefore, this study will supplement self-administered response by observation of actual performance by using observation check list. There is lack of evidence concerning nurses’ practice towards pressure ulcer prevention in Ethiopia and there is no study in the study area. Therefore, this study aimed to assess Pressure ulcer prevention practices and associated factors among nurses in public Hospitals of Eastern Ethiopia.

## Methods

### Study area, design and period

An institution based cross-sectional study was conducted in public hospitals of Harari Regional state and Dire Dawa city administration, Eastern Ethiopia from the 1^st^ February to the 1^st^ March, 2018. Harar is the capital city of Harari regional state, which is 523 Km away to East from capital city of Ethiopia, Addis Ababa. Based on 2007 census conducted by Central Statistical Agency of Ethiopia (CSA), Harari region has a total population of 183,415 of whom 92,316 were male [19]. In Harar there are five Hospitals. This study was conducted in three government hospitals. Hiwot Fana Specialized University Hospital (HFSUH) is a teaching hospital of Haramaya University with a total of 161 beds. Jugal Hospital (JH) is a regional referral hospital of Harari regional state with 95 beds. There are a total of 363 nurses working in governmental hospitals of Harari regional state.

Dire Dawa City Administration located in the eastern part of the country at a distance of 515 km from the capital city. According to the 2007 Census, Dire Dawa had a total population of 341,834, of whom 170,373 women [19]. Currently, there are two government hospitals in this city; Dilchora referral hospital and Sabian Primary hospital. Dilchora is a referral hospital of Dire Dawa city administration with a total of 190 nurses and Sabian Primary Hospital is also another Hospital of Dire Dawa city administration with a total of 60 nurses.

### Study population

All nurses working in Harari regional state and Dire Dawa city administration public hospitals were considered as the study population for this study. Nurses who were working for at least six months and available during data collection period were included. Those nurses who didn’t work in wards through rotation before and during the study period were excluded.

### Sample size determination

The sample size was calculated using single population proportions using the assumption of 95% CI with 5% margin of error, and double population formula using Epi Info Version 7 for individual factors using the assumption of 80% power and 1:1 ratio of exposed to non-exposed. The calculated sample size was 384 and after adding 10% non-response rate the final sample size became 422.

### Sampling procedure and sampling technique

**Fig 1:**
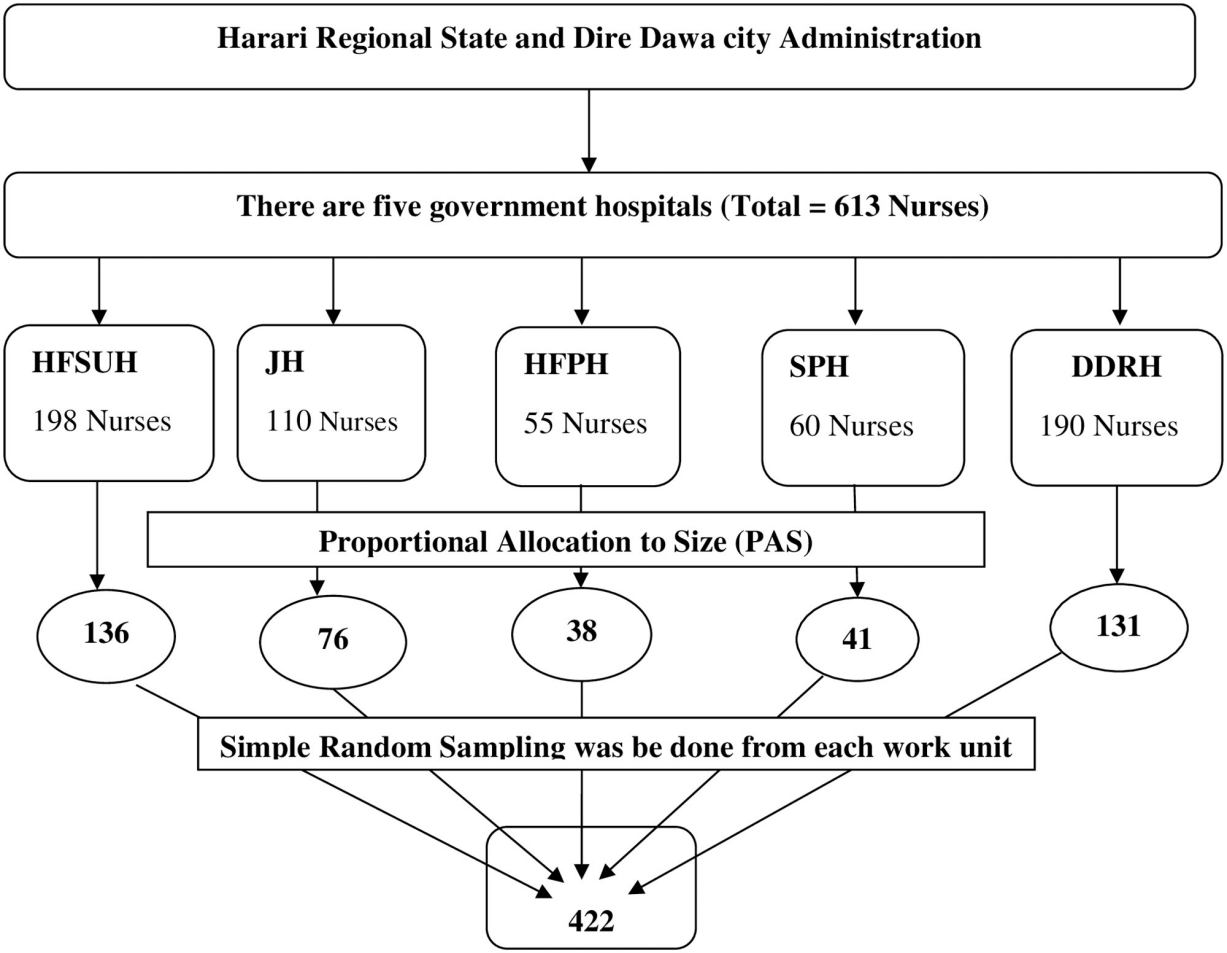
Schematic diagram of the sampling procedure for the study to assess pressure ulcer prevention practice and associated factors among nurses working in public hospitals of Eastern Ethiopia, 2018.

The sample for both observational checklist and self-administered questionnaire were taken by proportional allocation for each five public hospital and the individual participant was selected by simple random sampling for the self-administered questionnaire and for the observation the participants were selected purposively among those nurses assigning to bed ridden patients or patients at risk for pressure ulcer (Fig 1).

### Data collection tools

The data were collected from the study individuals using a pretested structured self-administered questionnaire and observational checklist. English version questionnaire was used for both the interview and observational checklist. The data collection tool was developed by reviewing different literature and consisted of five parts **Part I**: A socio demographic data, **Part II**: Knowledge level of the respondent (15 items), which was developed by reviewing previous articles [16, 20–22], **part III**: Questions on practice of pressure ulcer prevention (8 items), **Part IV**: Job satisfaction of the respondent (14 items), it was adopted from Job Satisfaction Survey (JSS) [23], and part **V**: Questions on factors associated with pressure ulcer prevention practice.

Observation checklist was adapted from previous study (18 items) related to prevention of occurrence of PU while nurses giving pressure ulcer prevention care to the bed ridden or at risk patients. Each item was then categorized as ‘done’ and ‘not done’ [8, 16, 18].

### Data collection procedures and data collectors

Five diploma trained nurses facilitated the data collection and two BSC nurses supervised the data collection. Data were collected through structured self-administered questionnaire and observational checklist. The non-participatory observation was done prior to distributing self-administered questionnaire and oral consent was given for those nurses who were going to be observed. Observation using observational checklist was done for 10% of the study population to assess the actual pressure ulcer prevention practice among nurses who were assigned to patients identified at-risk for pressure ulcer or bed ridden and used as a supplement to a study.

### Data quality control

Training was given for data collectors and supervisors regarding to objectives, questionnaires, checklist and ways of conducting the data collection. Before the actual data collection, pretest was conducted in 5% of sample size. After pretest any ambiguity, confusions, difficult words and differences in understanding were revised. Completeness and consistency of questionnaire were checked before and immediately after data was collected by each data collectors and supervisors. Double data entry was done by two data clerks and consistency of the entered data was cross checked by comparing the two separately entered data.

### Data processing and analysis

The collected data were cleaned, coded, and entered in to Epi Data 3.3.1 statistical software package. The statistical analysis was done using SPSS version 22. Frequency distribution for selected variables was done. The statistical significance and strength of the association between independent variables and an outcome variable were measured by the bivariate logistic regression model. For analysis of the outcome variable, practice of pressure ulcer prevention, the mean and above was coded as” 1” and below the mean was coded as” 0”. A variable P-value less than 0.2 was candidates to multivariable logistic regression model and a p-value less than 0.05 was considered as significantly associated. Finally, the results of the study were presented using tables, figures, and texts based on the data obtained. Descriptive statistics was carried out for the observation of pressure ulcer prevention practice of nurses and percentage of done was calculated and used.

### Operational definitions

#### Good knowledge

Nurses, who scored the correct answers for knowledge related questions’ regarding PUP above or equal to the mean value were considered to have good knowledge [16].

#### Job satisfaction

A worker who have scored above or equal to the mean score were considered to have job satisfaction [24].

#### Bedridden patients

Are patients who are unable to move out of bed or confined to bed due to old age, physical impairment, mobility problems, illness or injury or arising from medical restriction to ambulate [25].

### Ethical approval and consent to participant

Officially written approval letter was obtained from the Institutional Health Research Ethical Review Committee (IHRERC) of the College of Health and Medical Sciences, Haramaya University. Besides, an official letter was issued from the College of Health and Medical Sciences, Haramaya University to the director of each hospital. After securing permission from each hospital administrator, the actual data collection and observation was commenced after obtaining written and signed voluntary consent from each study participant. All information collected from the participants was kept confidential.

## Results

### Socio-demographic characteristics

In this study, a total of 401 study participants were involved, with a response rate of 95.02%. From the total number of respondents, more than half 237 (59.1%) were males and the mean age of the respondents was 29.63 + 6.67. Regarding educational status nearly three-fourth of the respondents 306 (76.3%) were BSC and above degree holders in nursing with a mean work experience of 6.42+ 5.509 (Table 1).

**Table 1:** Socio-demographic characteristics of nurses working in government hospitals of Harari regional state and Dire Dawa city administration, Eastern Ethiopia, 2018 (n = 401).

Variables	Category	Frequency	Percentage
**Sex**	Male	237	59.1
	Female	164	40.9
**Age in year**	18–27	178	44.4
	28–37	173	43.1
	38–47	35	8.7
	≥48	15	3.7
**Marital Status**	Single	200	49.9
	Married	192	47.9
	Divorced	9	2.2
**Religion**	Orthodox	233	58.1
	Muslim	116	28.9
	Protestant	44	11
	Catholic	3	0.7
	Other	5	1.2
**Ethnicity**	Oromo	188	46.9
	Amhara	169	42.1
	Tgrie	25	6.2
	Other	19	4.7
**Educational status**	Diploma	95	23.7
	BSc nurse &above	306	76.3
**Year of experience in completed years**	< 5	214	53.4
	6–10	139	34.7
	>10	48	11.7

### Knowledge about pressure ulcer

**Fig 2:**
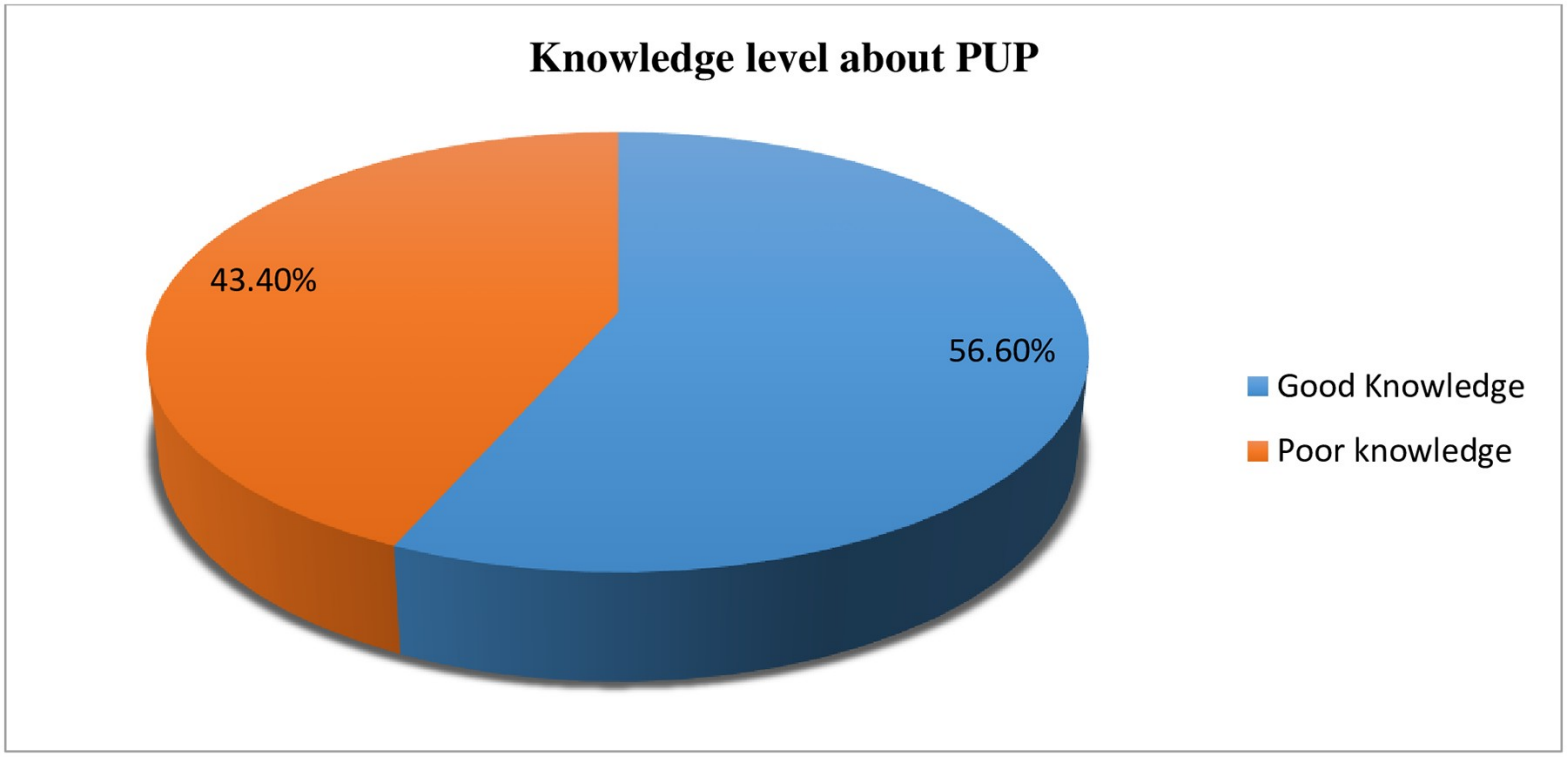
Nurses knowledge about pressure ulcer prevention in public hospitals of Harari regional state and Dire Dawa city administration, Eastern Ethiopia, 2018 (n = 401).

More than half 227 (56.6%) of the study participants were found to had a good knowledge about pressure ulcer prevention methods. Among nurses involved in this study, less than one-fifth 44(11%) of nurses were trained on pressure ulcer prevention. Regarding job satisfaction, nearly half 198 (49.4%) of the respondents were satisfied with their job (Fig 2).

### Work environment and patient related characteristics

Regarding the ward distribution, nearly one-fifth (21.9%) of the respondents was working in medical ward, followed by gynecologic/obstetrics ward (20.4%) and surgical ward (20%). Nearly one- third, 139 (34.7%) of nurses were reported that the presence of guideline for pressure ulcer prevention practice. More than one fourth (27.2%) of nurses reported as they had pressure reliving device in their working area. From the total study participants, 244(60.8%) had work load. In this study, more than half 213(53.1%) of the respondents informed that patients were not cooperate in PUP care in their working area (Table 2).

**Table 2:** Work environment and patient related characteristics of study participants in public hospitals of Harari regional state and Dire Dawa city administration, Eastern Ethiopia, 2018 (n = 401).

Variable	Category	Frequency	Percent
**Working unit**	Medical	88	21.9
	Surgical	80	20
	Orthopedics	26	6.5
	Gynecologic/obstetrics	82	20.4
	ward	41	10.2
	ICU	65	16.2
	OPD	19	4.7
**Presence of guide line for PUP**	Yes	139	34.7
	No	262	65.3
**Availability of PRD**	Yes	109	27.2
	No	291	72.8
**Workload**	Yes	244	60.8
	No	157	39.2
**Team work among staff**	Yes	267	66.6
	No	134	33.4
**Patient cooperativeness in PUP care**	Yes	188	46.9
	No	213	53.1

### Pressure ulcer prevention practice

More than half of the study participants 215(53.6%) were always performing skin care as a routine work, while 206(51.4%) and 202(50.4%) nurses sometimes maintain the head of the bed at or below 30-degree and advised care givers to use cream respectively. Nearly one-third of the respondents, 155(38.7%) reported that they had never used a risk assessment scale to assess pressure ulcer risk and 159(39.7%) nurses sometimes used a risk assessment scale (Table 3).

**Table 3:** Pressure ulcer prevention practice among nurses working in public hospitals of Harari regional state and Dire Dawa city administration, Eastern Ethiopia, 2018 (n = 401).

Nurses practice on pressure ulcer prevention	Rate of nurse’s practice					
	Never		Sometimes		Always	
	No	%	No	%	No	%
Performing skin assessment	96	23.9	196	48.9	109	27.2
Using assessment scale	155	38.7	159	39.7	87	21.7
Documenting all data related to PU assessment	89	22.2	172	42.9	140	34.9
Assess and provide pain management	53	13.2	135	33.7	213	53.1
Using pillows or foam wedges between bony prominences.	95	23.7	181	45.1	125	31.2
Placing water filled glove under the patient’s leg	131	32.7	178	44.4	92	22.9
Using or advising care givers to use creams or oils	94	23.4	202	50.4	105	26.2
Using absorbent pads or diapers that wick and hold moisture	95	23.7	188	46.9	118	29.4
perform skin care as a routine work	56	14.0	130	32.4	215	53.6
Encouraging and providing nutrition and fluids for malnourished patient as ordered	58	14.5	134	33.4	209	52.1
Monitoring patient’s intake and out put	62	15.5	158	39.4	181	45.1
Maintaining the head of the bed at or below 30-degree	50	12.5	206	51.4	145	36.2
Using of lift sheets or lifts equipment during transfer and position changes.	94	23.4	180	44.9	127	31.7
Turning patient every two hours	73	18.2	178	44.4	150	37.4
Bed making and maintain the bed linens are clean, dry and wrinkle free at all times.	49	12.2	153	38.2	199	49.6
Providing back massage.	90	22.4	189	47.1	122	30.4
Giving advice for patient or care giver regarding pressure ulcer prevention	57	14.2	167	41.6	177	44.1
Avoid massaging bony prominences	110	27.4	174	43.4	117	29.2

**Fig 3:**
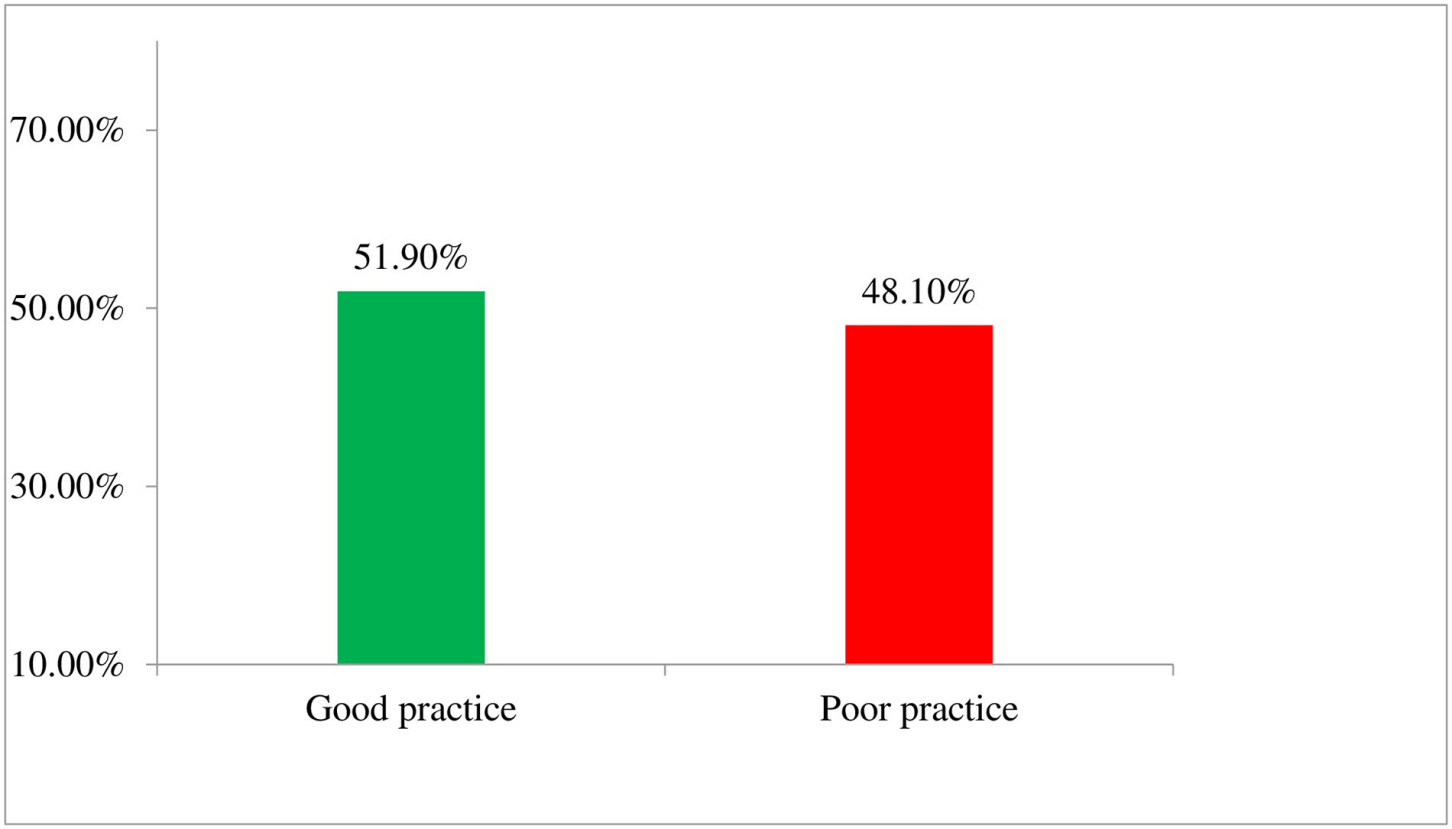
Pressure ulcer prevention practice level among nurses working in public hospitals of Harari regional state and Dire Dawa city administration, Eastern Ethiopia, 2018.

Overall, 208 (51.9%) (95% CI: 47.1%, 56.4%) of nurses reported that they have good pressure ulcer prevention practice (Fig 3).

### Results from observation checklist

To strengthen the findings from the interview obtained through questionnaire, observation was done by using observation check list that included medical, surgical, ICU and orthopedic ward in each hospital. Out of 42 observed participants, majority 35 (83.3%) of them were assessed and provide pain management for patients who experience pain. All of the nurses observed during study period were not using an assessment tool to assess pressure ulcer risk and majority (97.6%) did not re-position patients at risk every two hours. In general from observational findings, the proportion of nurses who were practicing proper pressure ulcer prevention practice was 19 (45.2%) (Table 4).

**Table 4:** Observation checklist for pressure ulcer prevention practice among nurses working in public hospitals of Harari regional state and Dire Dawa city administration, Eastern Ethiopia, 2018 (n = 42).

Observation Items	Frequency	
	Done	Not done
Performing skin assessment	18(42.9)	24(57.1)
Using assessment scale	0	42(100)
Documenting all data related to PU assessment	15(35.7)	27(64.3)
Assess and provide pain management	35(83.3)	7(17.7)
Using pillows or foam wedges between bony prominences.	2(2.8)	40(95.2)
Placing water filled glove under the patient’s leg	4(9.5)	38(90.5)
Using or advising care givers to use creams or oils	6(14.3)	36(85.7)
Using absorbent pads or diapers that wick and hold moisture	12(28.6)	30(71.4)
Performing skin care	17(40.5)	25(59.5)
Encouraging and providing nutrition and fluids for malnourished patient as ordered	33(78.6)	9(21.4)
Monitoring patient’s intake and out put	27(64.3)	15(35.7)
Maintaining the head of the bed at or below 30-degree	29(69.05)	13(30.95)
Using of lift sheets or lift equipment during transfer and position changes.	7(16.7)	35(83.3)
Turning patient every two hours	1(2.4)	41(97.6)
Bed making and maintain the bed linens are clean, dry and wrinkle free.	21(50)	21(50)
Providing back massage.	3(7.1)	39(92.9)
Giving advice for patient or care giver regarding pressure ulcer prevention	6(14.3)	36(85.7)
Avoid massaging bony prominences	5(11.9)	37(88.1)
Over all PUP practice, Good, Poor	19	45.2
	23	54.8

### Factors associated with pressure ulcer prevention practice

Bivariate analysis results showed that educational qualification level of the nurse, work experience, training, availability of pressure reliving device in working area, presence of pressure ulcer prevention guideline, work load, knowledge and satisfaction level of nurses were significantly associated with pressure ulcer prevention practice.

Variables with a p value of <0.2 in the bivariate analysis were candidate for multivariable analysis. In multivariable logistic regression analysis, educational qualification level, availability of pressure reliving device, knowledge and satisfaction level of nurses’ were identified to be significantly associated with practice of pressure ulcer prevention.

Nurses who had bachelor degree & above were nearly two times more likely to have good practice towards prevention of pressure ulcer as compared to those nurses who had diploma. Availability of pressure reliving device with in the hospital was associated with nurses’ pressure ulcer prevention practice, those nurses who responded to have Pressure ulcer prevention equipment were two times more likely to have good practice towards prevention of pressure ulcer than the counterpart. Those participants who had good knowledge about pressure ulcer prevention were 2.3 times more likely to have good practice when compared to those who had poor knowledge. Nurses who were satisfied with their job were nearly two times more likely to have good practice of pressure ulcer prevention than the counterpart (Table 5).

**Table 5:** Bivariate and multivariable analysis result for factors associated with pressure ulcer prevention practice among nurses working in public hospitals of Harari regional state and Dire Dawa city administration, Eastern Ethiopia, 2018 (n = 401).

Variables	Category	Practice level		COR (95% CI)	AOR (95% CI)
		Good	Poor		
Educational qualification	Diploma	38	57	1.00	1.00
	Bsc & Above	170	136	1.87 [1.17, 2.99]*	1.7 [1.02, 2.83]*
Year of experience	< 5 years	106	108	1.00	1.00
	6–10 years	78	61	1.3 [0.84, 2]*	1.32 [0.84, 2.08]
	> 10 years	24	24	1.02 [0.54, 1.91]	1.15 [0.59, 2.24]
Presence of guide line for PUP	Yes	79	60	1.00	1.00
	No	129	133	0.74 [0.48, 1.11]	0.74 [0.46, 1.19]
Availability of PRD	Yes	72	37	2.23 [1.41, 3.53]**	2.2 [1.34, 3.63]*
	No	136	156	1.00	1.00
Training on PUP	Yes	28	16	1.00	1.00
	No	180	177	0.58 [0.3, 1.11]	0.88 [0.43, 1.78]
Knowledge level about PUP	Good	139	88	2.4 [1.6, 3.6]**	2.3 [1.48, 3.55]**
	Poor	69	105	1.00	1.00
Job Satisfaction level	Satisfied	116	82	1.71 [1.15, 2.53]*	1.65 [1.09, 2.52]*
	Not satisfied	92	111	1.00	1.00
work load	Yes	117	127	0.67 [0.45, 1]	0.7 [0.45, 1.07]
	No	91	66	1.00	1.00

* = p-value <0.05,

** = p-value <0.001, CI = Confidence Interval, COR = Crude odds Ratio, AOR = Adjusted Odds Ratio.

## Discussion

The finding of this study showed that 51.9% (95% CI: 47.1%, 56.4%) of nurses had good pressure ulcer prevention practice. On observation, the proportion of nurses who were practicing proper pressure ulcer prevention practice was 45.2%. Pressure ulcer prevention practice, were significantly associated with current educational qualification of nurses, availability of pressure reliving device, job satisfaction and pressure ulcer related knowledge.

The prevalence of self-reported practice of nurses in this study is in line with the results of the studies conducted in India (48.2%) and Gondar University Hospital; Northwest Ethiopia (48.4%) [3, 6]. The finding of this study was higher than studies conducted in United Arab Emirates (10.3%) and Egypt (6.6%) [16, 26]. This difference may be due to difference in availability of resources, settings in which data collection was done, study population, the study design used and measurement criteria to categorize nurses’ level of practice. On the contrary the finding of this study was lower than a study conducted in Addis Ababa (67.3%) [27]. This discrepancy might be due to deference in knowledge of the nurses concerning prevention of pressure ulcer.

From this current study what nurses mostly never do is using assessment scale to assess pressure ulcer 38.7%95%CI (33.4%, 43.6%). This was also supported with observational study, All, of the nurses observed during study period were not use any assessment tool to identify patients with at risk of pressure ulcer. This might be due to lack of evidence based nursing practice and in-service training on prevention of pressure ulcer.

This study showed that nurses who had bachelor degree and above were nearly two times more likely to have good practice towards prevention of pressure ulcer as compared to those nurses who had diploma. This is in line with studies conducted in Spain, Jordan and Korea [26–28]. This might be due to increasing educational level, nurses may able to understand and employ a risk assessment tool in a better way than that of diploma graduates. In addition, it could also be due to the basic knowledge and in-depth training received during academic years, which is different than that received by diploma nurses. Because bachelor degree and above nurse’s education is more comprehensive, they have a deeper knowledge base to draw on in areas such as clinical practice and critical thinking.

The odd of good practice of pressure ulcer prevention was 2.3 times higher among nurses with good knowledge of pressure ulcer prevention than the counterpart. This finding is similar with studies conducted in Ethiopia and Egypt showed that there were positive relationship between knowledge and practice of the nursing staff regarding pressure ulcer prevention [6, 29, 30]. This might be due to the fact that good knowledge improves the confidence and readiness of nurses to perform their routine activities.

In this study availability of sufficient pressure-relieving devices or equipment was an independent predictor for nurse’s pressure ulcer prevention practice. This is supported by different studies which found that Scarcity of pressure reliving equipment was the identified factors which limit nurses’ practice of pressure ulcer prevention [16, 31, 32]. This might be due to the fact that adequate access to sufficient equipment may encourage nurse’s motivation and ability to prevent patients from developing pressure ulcer. The availability of sufficient equipment in the workplace plays a key role in facilitating care delivery, decrease in stress, minimized delay to care, and patient satisfaction.

In this study job satisfaction was nearly two times more likely to have a good pressure ulcer prevention practice than the counterpart. This finding was consistent with studies conducted in different part of Ethiopia in which nurses who satisfied with their job were more likely to have good pressure ulcer prevention practice [6, 16]. This could be due to the fact that when the nurses satisfied with their job, they could experience meaningfully, greater responsibility, and better use of their knowledge and skills in their job and such situation leads to be motivated in their work to apply all their knowledge and experiences on practices related to prevention of pressure ulcer. It might also due to the fact that, since they are satisfied with their job, they might be eager in helping and caring for patients.

The findings of this study were interpreted cautiously in light of some limitations. The use of a cross-sectional survey design did not allow for the generalization of the findings beyond the sample from which data was gathered. The collected data was based on self-report, and observational check list, therefore, the finding may not be consistent. The data was collected only from nurses and observation was done while the have given care for the respondents, but there are other predictors like nutritional status which can be collected from the respondents and can be an independent factors for pressure ulcer. Therefore in this study some independent factors which can be contributed for pressure ulcer was not be incorporated. Despite these limitations, this study has provided the foundation for future empirical studies among nurses who are working in health institutions and the clinical practice can be improved based on the findings. Future studies may use qualitative design to understand and address the key drives why the pressure ulcer prevention practice was low.

## Conclusions

In this study, more than half of nurses were reported that they had good practice towards pressure ulcer prevention practice. Educational qualification level, Availability of PU relieving devices, being satisfied with their job and having good knowledge about pressure ulcer prevention were found to be independent predictors for good pressure ulcer prevention practice. Nurses should provide patient centered care and show commitment in applying pressure ulcer prevention methods to improve the quality of nursing care. They should update their knowledge on pressure ulcer prevention both in theoretical as well as practical aspect and those who had better knowledge should also teach their respective colleagues who had deficits for the improvement of nursing care.

The hospital administrators should strive for preventing occurrence of pressure ulcer through training and educating nurses, monitoring compliance and providing feedback, and embedding the practice of pressure ulcer prevention in the institutional safety culture and patient engagement. Researchers can do further investigation to identify other factors by using other tools and study design.

## Supporting information

S1 File (DOCX)

## Abbreviations

DRH: Dil Chora Referral Hospital
EBP: Evidence Based Practice
HFSUH: Hiwot Fana Specialized University Hospital
ICU: Intensive Care Unit
IHRERC: Institutional Health Research Ethics Review Committee
JH: Jugal Hospital
NPUAP: National Pressure Ulcer Advisory Panel
PRD: Pressure reliving device
PU: Pressure Ulcer
PUP: Pressure Ulcer Prevention
