# Supplementary material for: Pressure ulcer prevention practices and associated factors among nurses in public hospitals of Harari regional state and Dire Dawa city administration, Eastern Ethiopia
Source: PLoS One. 2020 Dec 15;15(12):e0243875. doi: 10.1371/journal.pone.0243875 (PMC7737888; doi:10.1371/journal.pone.0243875)
Supplement: S1 File — (DOCX) [file pone.0243875.s001.docx]

## Data collection tool

| **Part I: Socio-demographic characteristics of the respondents.**  Instruction: Please circle to the number which contain your correct answer (the most appropriate answer) or in the space provided. | | | Code | Skipping pattern |
| --- | --- | --- | --- | --- |
| **S/NO.** | Questions | Responses |  |  |
| 101 | How old are you? | _____ (in completed yrs.) |  |  |
| 102 | What is your Sex? | 1. M 2. F |  |  |
| 103 | What is your marital status? | 1. Single 2. Married 3. Divorced 4. Widowed |  |  |
| 104 | What is your Religion? | 1. Orthodox 2. Muslim 3. Protestant 4. Catholic 5. Other (specify) ________ |  |  |
| 105 | What is your ethnicity? . | 1. Oromo 2. Amhara 3. Tigre 4. Other (Specify) _______ |  |  |
| 106 | What is your current highest educational level? | 1. Diploma 2. BSc.N. 3. Masters |  |  |
| 107 | How long is your experience in nursing profession? | _________ Yrs. |  |  |

| **Part II: Knowledge about pressure ulcer prevention**  Please read each statement carefully and tick the mark sign“√” in the corresponding column that most likely reflects your answer to the following questions. | | | | | | | | | | |
| --- | --- | --- | --- | --- | --- | --- | --- | --- | --- | --- |
| **S/NO.** | **Questions/items** | | | | **True** | | **False** | | **I don’t know** | |
| 201 | Risk factors for development of pressure ulcers are immobility, incontinence, impaired nutrition and altered level of consciousness. | | | |  | |  | |  | |
| 202 | Hot water and soap may dry the skin and increase the risk for pressure ulcers. | | | |  | |  | |  | |
| 203 | It is important to massage bony prominences. | | | |  | |  | |  | |
| 204 | The first sign of pressure ulcer development is open sore | | | |  | |  | |  | |
| 205 | All individuals should be assessed on admission to a hospital for risk of pressure ulcer development. | | | |  | |  | |  | |
| 206 | Partial skin loss with blister and abrasion is correct answer for the sign of stage II pressure ulcer. | | | |  | |  | |  | |
| 207 | A Braden scale is a risk assessment tool used for assessing pressure ulcer. | | | |  | |  | |  | |
| 208 | Patient skin should be clean and dry to prevent risk of pressure ulcer development. | | | |  | |  | |  | |
| 209 | Heel ulcer is prevented by putting pillow under the patient’s leg. | | | |  | |  | |  | |
| 210 | For bed ridden and patient who has incontinence, skin cleaning should occur at the time of soiling and at routine intervals. | | | |  | |  | |  | |
| 211 | Vitamin C & E are important to maintain skin integrity. | | | |  | |  | |  | |
| 212 | Serum albumin test is the appropriate laboratory test for nutritional assessment of pressure ulcer patient. | | | |  | |  | |  | |
| 213 | Turn position for every 2 hours is significant activity for protecting skin damage for bed ridden patients. | | | |  | |  | |  | |
| 214 | Lift up the patient without dragging is a correct practice for maintaining skin integrity | | | |  | |  | |  | |
| 215 | Topical cream is appropriate method for skin care | | | |  | |  | |  | |
| **Part III: Self-reported Practice of pressure ulcer prevention**  Please read each statement carefully and tick the mark sign“√” in the corresponding column that most likely reflects your answer to the following questions. | | | | | | | | | | |
| **S/NO.** | **Questions/items** | | | | **Never** | | **Sometimes** | | **Always** | |
| 301 | I do a skin assessment for pressure ulcer. | | | |  | |  | |  | |
| 302 | I use a risk assessment scale to assess pressure ulcer risk | | | |  | |  | |  | |
| 303 | I document all data related to pressure ulcer Assessment | | | |  | |  | |  | |
| 304 | I assess and provide management of pain in the patients who experience pain from any causes. | | | |  | |  | |  | |
| 305 | I use pillows or foam wedges to avoid contact between bony prominences. | | | |  | |  | |  | |
| 306 | I use water filled glove under the patient’s leg | | | |  | |  | |  | |
| 307 | I use or advice care giver to use creams or oil on patient’s skin in order to protect from urine, stool or wound drainage. | | | |  | |  | |  | |
| 308 | I use absorbent pads or diapers that wick and hold moisture away from the skin. | | | |  | |  | |  | |
| 309 | I perform skin care as a routine work of my unit. | | | |  | |  | |  | |
| 310 | I Encourage and provide nutrition and fluids for patients who are malnourished. | | | |  | |  | |  | |
| 311 | I Monitor patient’s intake and output (I&O). | | | |  | |  | |  | |
| 312 | I Maintain the head of the bed at/or below 30-degrees, if consistent with patient’s medical condition. | | | |  | |  | |  | |
| 313 | I use lift sheets or lift equipment to avoid dragging clients during transfer and position changes. | | | |  | |  | |  | |
| 314 | I turn a bed ridden patient position every two hours | | | |  | |  | |  | |
| 315 | I Make bed making and maintain the bed linens are clean, dry and wrinkle free at all times. | | | |  | |  | |  | |
| 316 | I provide frequent back massage | | | |  | |  | |  | |
| 317 | I give advice to patient and caregiver regarding prevention of pressure ulcer. | | | |  | |  | |  | |
| 318 | I avoid massage over patient’s bony prominences to Pressure ulcer formation. | | | |  | |  | |  | |
| **Part IV: Job satisfaction survey (JSS):**  Please read each statement carefully and tick the mark sign“√” in the corresponding column that most likely reflects your answer to the following questions | | | | | | | | | | |
| **S/NO.** | **Questions/items** | **Disagree very much** | **Disagree Moderately** | **Disagree slightly** | | **Agree slightly** | | **Agree Moderately** | | **Agree very Much** |
| 401 | I Feel being paid a fair amount for the work I do |  |  |  | |  | |  | |  |
| 402 | I Feel satisfied with my chances for salary increase. |  |  |  | |  | |  | |  |
| 403 | There is really too little chance for promotion on my job. |  |  |  | |  | |  | |  |
| 404 | I am satisfied with my chances for promotion. |  |  |  | |  | |  | |  |
| 405 | My supervisor is unfair to me. |  |  |  | |  | |  | |  |
| 406 | When I do a good job, I receive the recognition for it that I should receive. |  |  |  | |  | |  | |  |
| 407 | There are few rewards for those who work here. |  |  |  | |  | |  | |  |
| 408 | Many of our rules and procedures make doing a good job difficult. |  |  |  | |  | |  | |  |
| 409 | I have too much to do at work. |  |  |  | |  | |  | |  |
| 410 | There is too much bickering and fighting at work |  |  |  | |  | |  | |  |
| 411 | I sometimes feel my job is meaningless. |  |  |  | |  | |  | |  |
| 412 | I like doing the things I do at work. |  |  |  | |  | |  | |  |
| 413 | Communication seems good within this hospital. |  |  |  | |  | |  | |  |
| 414 | Work assignments are not fully explained. |  |  |  | |  | |  | |  |

| **Part V: Other factors** | | | | |
| --- | --- | --- | --- | --- |
| **S/NO.** | **Questions** | | Responses | Skip |
| 501 | Which working area are you currently serving? | 1. Adult Medical ward 2. Adult Surgical ward 3. Orthopedic ward 4. Gynecologic and obstetrics ward 5. ICU 6. Others(specify)_________ | |  |
| 502. | Is their pressure ulcer prevention guideline available in your working unit? | 1. Yes 2. No | |  |
| 503 | Is there a pressure relieving device available in your working unit? | 1. Yes 2. No | | If2, skip  to no.505 |
| 504 | If yes, which one is available in your working unit? (You can choose more than one?) | 1. Elbow pad 2. Special mattress such as foam 3. Pillow 4. A bed cradle 5. Other(specify) _________ | |  |
| 505 | On average, how many patients do you care per shift (on daily bases)? | in number_____ | |  |
| 506 | Do other health workers involving in prevention and management of pressure ulcers in your working unit? | 1. Yes 2. No | |  |
| 507 | Do you get formal training about pressure ulcer prevention since qualified as a nurse? | 1. Yes 2. No | | If2, skip  to no.509 |
| 508 | If yes, when you had last received training? | _____________Yrs. | |  |
| 509 | Do you give higher priority for other illnesses than PU in your working unit? | 1. Yes 2. No | |  |
| 510 | Are the patients always able to cooperate in pressure ulcer prevention care in your working unit? | 1. Yes 2. No | |  |
| 511 | If no, what patient-related barriers you perceived made it difﬁcult for them to undertake PUP activities? | 1. The patients are too ill or medically unstable. 2. The patients are not interested in participating 3. The patients have a co-morbid diagnosis of dementia with high degree of aggression 4. Other (specify)_______ | |  |

## Part VI: Observation Checklist

Observational check list for the role of nurses on pressure ulcer prevention practice for patients identified at-risk for pressure ulcer or bed ridden.

| Checklist no.:_______ Hospital: 1. HFSUH 2. JH 3. HFPH. 4. DDRH. 5. SPH  Time of observation:_______ | | | |
| --- | --- | --- | --- |
| S no | Observation items | Done | Not done |
| 1 | Doing a skin assessment for pressure ulcer. |  |  |
| 2 | Using of a risk assessment scale to assess pressure ulcer risk |  |  |
| 3 | Documenting of all data related to pressure ulcer assessment (including what was observed and what actions were taken). |  |  |
| 4 | Assessing and providing management of pain in the patients who experience pain from any causes |  |  |
| 5 | Using of pillows or foam wedges to avoid contact between bony prominences. |  |  |
| 6 | Placing water filled glove under the patient’s leg |  |  |
| 7 | Using or advising care giver to use creams or protective moisture barrier ointment on patient’s skin to protect skin from urine, stool or wound drainage |  |  |
| 8 | Using of absorbent pads or diapers that wick and hold moisture away from the skin |  |  |
| 9 | Cleaning of the skin with sterile water, normal saline, or pH balanced skin cleanser |  |  |
| 10 | Encourage and provide nutrition and fluids as ordered. |  |  |
| 11 | Monitoring patient’s intake and output (I&O). |  |  |
| 12 | Maintain the head of the bed at/or below 30-degrees, if consistent with patient’s medical condition. |  |  |
| 13 | Use of lift sheets or lifts equipment during transfer and position changes. |  |  |
| 14 | Turning of patients at risk every two hours |  |  |
| 15 | Bed making and maintaining the bed linens clean, dry and wrinkle free. |  |  |
| 16 | Providing back massage. |  |  |
| 17 | Educating a patient or a caregiver about the prevention of pressure ulcers |  |  |
| 18 | Avoid massaging over patient’s bony prominences |  |  |
